# National Genome Initiatives in Europe and the United Kingdom in the Era of Whole-Genome Sequencing: A Comprehensive Review

**DOI:** 10.3390/genes13030556

**Published:** 2022-03-21

**Authors:** Jan Smetana, Petr Brož

**Affiliations:** 1 Institute of Food Science and Biotechnology, Faculty of Chemistry, Brno University of Technology, 61200 Brno, Czech Republic; 2 Department of Genetics and Molecular Biology, Institute of Experimental Biology, Faculty of Science, Masaryk University, 61137 Brno, Czech Republic; bbrozpetr@gmail.com

**Keywords:** national genome project, whole-genome sequencing, population, genetic variability Europe, United Kingdom

## Abstract

Identification of genomic variability in population plays an important role in the clinical diagnostics of human genetic diseases. Thanks to rapid technological development in the field of massive parallel sequencing technologies, also known as next-generation sequencing (NGS), complex genomic analyses are now easier and cheaper than ever before, which consequently leads to more effective utilization of these techniques in clinical practice. However, interpretation of data from NGS is still challenging due to several issues caused by natural variability of DNA sequences in human populations. Therefore, development and realization of projects focused on description of genetic variability of local population (often called “national or digital genome”) with a NGS technique is one of the best approaches to address this problem. The next step of the process is to share such data via publicly available databases. Such databases are important for the interpretation of variants with unknown significance or (likely) pathogenic variants in rare diseases or cancer or generally for identification of pathological variants in a patient’s genome. In this paper, we have compiled an overview of published results of local genome sequencing projects from United Kingdom and Europe together with future plans and perspectives for newly announced ones.

## 1. Introduction

The release of the first human reference genome in 2001 initiated the new era of approach into analyses of human genetic information [1]. Accomplishment of complete whole-genome sequences by pioneers of human genetics James D. Watson and Craig Venter a few years later opened a new path for the utilization of novel massive parallel (next-generation) sequencing [2,3]. These studies showed that 3 billion base pairs encode approximately 26,000 protein coding transcripts and that these coding transcripts represent only 1% of the whole genome. The beginning of the new millennia showed that there is a clear need for construction of reference genomes in order to unravel human genome variability between individuals.

Therefore, several sequencing projects, such as the International HapMap and later 1000 Genomes Project (1KGP), were launched to collect genetic data from various populations. Results from the cohort of 2504 individuals from 26 populations of a stage-three 1KGP project showed a total of nearly 88 million variants and led to the construction of the comprehensive catalogue of structural variants in human genome [4,5]. Data from HapMap and 1KGP were also used as the reference for several studies [6,7] and are still an essential part of several versions of a human reference genome. The current version is known as Genome Reference Consortium Human Build 38 patch release 13 (GRCh38.p13/hg38) [8].

Nowadays, the renaissance of genome-wide sequencing techniques allows us to perform genomic analyses more cheaply and effectively than ever before. The advantages of whole-genome sequencing (WGS) were highlighted in multiple genome-wide association studies (GWAS). The main aim of those studies is the testing of genetic variants across the genomes of many individuals in order to identify genotype–phenotype associations [9]. Data from such studies provided critical data for diagnostics of both rare or cancerous diseases, clinical counseling and decision making for utilization of eligible treatment protocol [10,11,12,13,14]. The vast majority of pathogenic mutations and SVs including deletions, duplications, and inversions were found in exons (coding parts of the genes). In addition, data from recent studies also showed that several disorders, such as Alzheimer’s disease or hemophilia A, can be caused by intronic splicing variants, which could have impact on the stability or regulatory function of mRNA [15,16,17]. Therefore, utilization of WGS technique and analysis of complex genomic data of the patient is considered one of the key steps for personalized medicine [18].

The aim of such analyses is usually to identify pathological or potentially pathological variants through multistep bioinformatic processing of sequencing data. Two major steps are data alignment (comparison between the “standard genome” and genetic information of the patients) and interpretation of genetic variants. Interpretation of genetic variants is commonly based on comparison of variants detected in patients with variants available through online databases such as GnomAD, ClinVar, HGMD, ClinGen or LOVD [19,20,21,22,23,24]. Comprehensive list of these databases is for example provided by the web pages of the Human Genome Variation Society (www.hgvs.org/locus-specific-mutation-databases). Online databases are broadly considered useful for annotation of genetic variants; nevertheless, there is still the need for better characterization of population-specific genetic variants and their potential significance in patients with rare diseases, neurodevelopmental disorders or in cancer patients treated by targeted therapy [25,26]. Many countries worldwide therefore started their own population-specific initiatives during the last decade. They are typically focused on description of local genetic variability using WGS, creation of online database of identified variants (“digital genome”) and possibly generation of population-specific genome assembly. In this review, we bring together basic information about national genome initiatives from the United Kingdom and Europe and discuss the potential novel projects and development in this field.

## 2. Materials and Methods

Google, PubMed, EGA (The European Genome-Phenome Archive), HGV (Human Genome Variation) and EMBL-EBI data archives were searched in September 2021 to gather information about national genomic initiatives by using the search parameters (<country name> [Title]) and (human genome project) OR (national genome initiative). Criteria for including into analysis were: (1) published results in PubMed, (2) functional website in English language with information about main specifics of the project (e.g., sequencing technology, cohort size), and (3) available information about funding and scientific board of the project. Studies not carried out in a European country were excluded from the analysis. Both authors overviewed the data. Discrepancies and/or inconsistencies were discussed and resolved through mutual agreement.

## 3. Results

### 3.1. United Kingdom

The UK has already initiated several genome-wide association studies (GWAS) in large cohorts of individuals. Apart from 1KGP, Wellcome Trust launched the UK10K project aiming at WGS and deep exome sequencing (80×) for identification of both rare and pathogenic SNPs and SVs in the UK population. The WGS cohort, “the UK10K-cohort arm”, analyzed data from 3781 individuals. The low pass WGS (median average read depth 7×) found 24 million variants overall, including over 3.5 million indels and 18,739 large deletions (median size 3.7 Kb). The genome of each individual contained on average 3,222,597 SNPs (5073 private), 705,684 indels (295 private) and 215 large deletions (less than 1 private). The dataset from the UK10K project is focused on the genotype/phenotype resource, which will be an order of magnitude deeper than the genetic-only 1000 Genomes Project dataset for Europe [27].

In 2012, National Health Services (NHS) has initialized a new era of genomic medicine with the 100,000 Genomes Project (https://www.genomicsengland.co.uk; accessed on 10 January 2022). Under the auspices of former Prime Minister David Cameron and as the part of the GBP 300 million initiative, NHS-owned company Genomics England is responsible for sequencing of 100,000 genomes from NHS patients with cancer and rare infectious diseases [28]. The project is focused on the better understanding of linkage between diseases and genetic signatures, potential application of genetic information in personal medicine and implementation of WGS into routine medical care [29]. For cancer, 50,000 genomes from 25,000 individuals (germline and tumor pairs) are expected to be collected. The other half of the genomes will involve 15,000 genomes of rare disease patients and 35,000 genomes of their relatives (mainly parents). Sequencing capacity and generation of sequencing data is covered by Illumina, while data analyses and interpretation are realized by several sub-contractors including Iceland’s WuXi NextCODE or Wellcome Trust Sanger Institute spin-off Congenica [30]. Nowadays, there are over 92,000 genomes sequenced and pilot studies from the project already showed important genomic data associated with leukemia [31] and rare diseases [32,33]. In addition, a new online database of genetic variants Human Genome Variation Archive (HGVA) was announced [34].

### 3.2. Iceland

Reykjavik’s company deCODE have gathered medical and genotypic data from Iceland’s population since 1996. The company researchers have already sequenced a considerable part of Iceland’s population. In their recent study, they sequenced genomes of 15,220 Icelanders using Illumina HiSeq platforms with median average read depth of 34x. Overall, they found a total of 31,079,378 SNPs and 7,940,790 indels. Known for its thorough genealogical datasets, they also described the parent of origin of 42,961 de novo mutations [35]. In comparison to other populations, the Icelandic population showed less rare variants and higher frequency of deleterious variants due to the limited population size and geographical isolation leading to higher influence of founder effect [36].

### 3.3. Sweden

The genetic map of Sweden population was already described in the SweGen study. Based on SNP genotyping of 10,000 individuals, samples from 1000 Swedes reflecting the genetic structure of the Swedish population were carefully selected for WGS [37]. Using a HiSeq X platform, they reached a median average read depth of 36× and found a total of 29.2 million SNPs and 3.8 million indels with 9.9 million of these variants not known in current databases. Furthermore, an average of 7199 individual-specific SNPs and 8645 larger SVs were observed per each sample. In addition, WGS data also showed genetic diversity within Sweden’s population (particularly between southernmost and northernmost population of the country) compared to other continental European populations. AS an output of SweGen study, SweFreq online database (https://swefreq.nbis.se/; accessed on 10 Januray 2022) was established, containing whole-genome variant frequencies of all 1000 sequenced Swedish individuals [38].

### 3.4. Finland

Finland is well-known for its population survey established in 1972 called FINRISK, which collects samples of 6000–8000 individuals every five years to study risk factors of chronic diseases in Finland [39]. Due to Finland’s unique population history and advantage resulted from FINRISK clinical data, there are several GWAS studies ongoing in Finland. Sequencing Initiative Suomi in Finland (SISU) compared exome sequence data of 3000 Finns to the same number of non-Finnish Europeans. Results from this recent SISU study showed that the Finnish gene pool has unique genetic features including fewer variable sites in genome, more low-frequency loss-of-function variants and almost twice as many low-frequency complete gene knockouts [40]. In 2017, the EUR 59 million FinnGen project (https://www.finngen.fi/; accessed on 12 January 2022) was launched as an academic-pharma consortium that involves nine Finnish biobanks, all Finnish University Hospitals and their respective Universities, the Institute of Health and Welfare (THL) and seven large pharmaceutical companies (Abbvie, AstraZeneca, Biogen, Celgene, Genentech, Merck/MSD and Pfizer). The aim is to obtain WGS data from 500,000 Finns, which enables ambitious study designs to improve understanding of the genetic background of diseases and, subsequently, implementation of genome medicine in clinical practice and drug development. There are already 200,000 existing legacy samples, mainly from the THL Biobank, and 300,000 additional prospective samples will be collected by all of the six Finnish hospital biobanks and the Blood Service’s biobank [41].

### 3.5. Denmark

The Denmark population-specific database of SNPs was based on data obtained from the “Danish pan-genome” study, in which authors used WGS for detailed analysis of genomes from 30 trios (parents-offspring). They reported 536,000 novel SNPs and 283,000 novel short indels detected by deep WGS (average read depth of 50×) and they develop a population-wide de novo assembly approach to identify 132,000 novel indels larger than 10 nucleotides with low false discovery rates [42]. Recently, a trio-based approach was utilized to create de novo assemblies of 150 individuals (50 trios) from GenomeDenmark project as a regional reference genome. This approach is unbiased against discovery of SVs and variation in the most complex parts of the genome, and it has the potential to improve the power of future association mapping studies [43].

### 3.6. Norway

The Norwegian 1000 genomes project was founded by the Norwegian Cancer Genomics Consortium (NCGC). While still in the process of collecting samples and processing samples, there is a working database of genetic variants, which already contains 1,547,121 individual variants acquired from 1590 normal chromosomes of cancer patients [44].

### 3.7. Estonia

The Estonian Genome Center of the University of Tartu (EGCUT) together with Estonian Biobank are collecting samples intended for GWAS studies in Estonian population. This study already consists of 51,535 donors (≥18 years of age), collected to appropriately reflect the age, sex and geographical distribution of the Estonian population (http://www.geenivaramu.ee/for-scientists/data-release/; accessed on 20 December 2021). WGS data are available from 100 individuals together with additional data from SNP arrays (20,000 individuals) and/or NMR metabolome data (11,000 individuals) [45].

### 3.8. Latvia

Since 2006, The Genome Database of the Latvian Population (LGDB) is collecting and processing health information, data, and biospecimens from representatives of the Latvian population. So far, the LGDB is comprised of samples and associated phenotypic and clinical information from 31,504 participants, constituting approximately 1.5% of the Latvian population [46].

### 3.9. Lithuania

Genetic data from Lithuanian population come from research project “Genetic diversity of the population of Lithuania and changes of its genetic structure related with evolution and common diseases” (acronym LITGEN). The group previously published data from SNP microarrays describing diversity and distribution of copy number variants (CNVs) in 286 unrelated individuals from the two main ethnolinguistic groups (Aukštaičiai and Žemaičiai) of the Lithuanian population [47]. Recently, first 96 exomes from healthy Lithuanian individuals were sequenced. An average of 42,139 SNPs and 2306 short indels were found in each individual exome together with five pathogenic genomic variants that were inherited in an autosomal recessive pattern and that statistically significantly differed from the European population data from 1KGP [48].

### 3.10. Spain

There are several smaller projects focused on genetic variability and rare diseases in the Spanish population, such as the Medical Genome Project (MGP) [49] or The Genoma 1000 Navarra Research Project (NAGEN 1000) [50]; however, a national genome project is missing. On the other hand, the CSVS (Collaborative Spanish Variability Server), a crowdsourcing database of the Spanish population genetic variability currently aggregates more than 2000 genomes and exomes of unrelated Spanish individual. Based on the collected data so far, CSVS produced the first version of the Spanish Genome Reference Panel (SGRP1.0) [51].

### 3.11. France

The French National Alliance for Life Sciences and Health (Aviesan) started in 2015 national plan: the EUR 670 million “2025 France Genomic Medicine Initiative (PFMG2025), responsible for introducing precision medicine into the care pathway and developing a national framework for “big-genomic data” medicine [52]. Technological aspects of the project are secured via France Genomique, an infrastructure which joins together the four main French public research organizations: CEA, CNRS, INRA and INSERM.

### 3.12. Netherlands

Genome of the Netherlands (GoNL) is a Dutch reference genome project in which whole genomes of 250 Dutch trios (750 individuals) were sequenced (average read depth of 13×) [53]. In 1990, the Netherlands also established a population-based cohort study called the Rotterdam study. Recently, 2628 DNA samples from this study were used for exome sequencing (average read depth of 53×) and this dataset was denoted “Rotterdam Study Exome Sequencing set 2” (RSX2). The authors of the projects have stated that from the 439,633 coding variants, 120,109 were absent from six other public population databases including ExAC2.0, ESP6500, 1 KG, Icelandic deCODE, GoNL and UK10K. The smallest overlap was seen with the Icelandic population, which is in line with previous statements. In general, each dataset showed contained variants not present in any of the other datasets. The results suggested that both smaller population-specific datasets as well as large aggregation datasets contributed information and each one of them contributed variants that were not seen yet [54].

### 3.13. Italy

In Italy, several GWAS studies characterized the genetic variability of local populations, including Sardinians [55] or Lombards in the North Italian region [56], using SNP-genotyping. In 2015, Italian National Commission for genomics was established in order to set up a national plan for the use of genomic knowledge and technologies in healthcare known as the Italian Genome project (IGP). Based on the sequencing data, a new Italian Genome Reference Panel (IGRP1.0) was defined. Pivotal results also extended the knowledge of genetic variability in the Italian population, including variants not known in the previous datasets such as β thalassemia-related variant GRCh37 chr11:g.5248004G>A (rs11549407), distribution of deleterious variants and incidence of human knockouts, and overall confirmed the necessity of distinct genome references for the Italian population [57].

### 3.14. Germany

A similar situation to France is found in Germany, where nowadays plenty of GWAS projects are running with the idea of building a human genome database; for example, a consortium consisting of Kühne-Stiftung, University Hospital Zurich (UHZ) and UKE Hamburg. This consortium plans to obtain whole-genome sequencing data from over 9000 people in the German-language area with funding of 12.5 million euros [58]. GWAS-suitable infrastructure is already in place in Germany, such as the German Human Genome-Phenome Archive (CHGA), which is available to scientific community through the German Cancer Research Center DKFZ, Heidelberg. In addition, The German Ministry of Health announced the foundation of the German Genomics Initiative (genomDE). GenomDE should entail a legal and ethical framework for organization, data infrastructure and reimbursement as well as a communication campaign aimed on both the public and healthcare professionals in Germany in the field of population genomics

### 3.15. Czech Republic

The National Center for Medical Genomics (NCGM) recently launched the project “Analysis of Czech Genomes for Theranostics” (ACTG), which is focused on WGS analysis of 1500 genomes from Czech population by the 2022. So far, the genomic database consists of 1055 analyzed genomes [59].

### 3.16. Poland

In Poland during 2014–2020, the European Centre for Bioinformatics and Genomics (ECBiG) consortium planned the sequencing of whole human genomes of about 5000 inhabitants from all over the country [60]. Pilot results from a cohort of 1079 individuals showed total of 31.24 million SNPs and 5.63 million small indels. On average, 4.48 million small variants per individual were found, of which 16,473 were private variants [61].

### 3.17. Slovenia

The Slovenian Genome project (SGP) was announced as support for the cooperation in +1MG. The consortium of University of Ljubljana, Institute of Oncology Ljubljana and Institute Service of Slovenia for Transfusion Medicine is focused on the creation of an environment for the collection of genetic, health and environmental risk factors and development of personalized medicine in Slovenia. The pilot project is based on sequencing of 300 Slovene genomes, which will represent a foundation of data analysis platform for the project [62].

### 3.18. Greece

The genomic initiative “Genome of Greece” (GoGreece) was launched in 2010 through The Laboratory of Pharmacogenomics and Individualized Therapy of the University of Patras. The project is based on WGS of >100,000 Greek individuals in order to delineate the genetic etiology of the underlying clinical phenotype of patients suffering from monogenic and multifactorial diseases and to determine the genetic variability of the Hellenic population [63]. Results from this cohort showed incidence of novel *FTO* and *TBC1D1* genetic variants associated with Amyotrophic lateral sclerosis (ALS) in the Greek population [64], or six genomic variants (*SLC9A4* c.1919G>A, *KIAA1109* c.2933T>C and c.4268_4269delCCinsTA, *HoxB6* c.668C>A, *HoxD12* c.418G>A, and *NCK2* c.745_746delAAinsG) with the potential of celiac disease predisposition in the Greek population [65].

### 3.19. Cyprus

The Cyprus genome initiative was funded as a part of EUR 38 million Horizont 2020 Biobanking and the Cyprus Human Genome Project, which is based on an existing Biobank of the University of Cyprus and its transformation to a Center of Excellence in Biobanking and Biomedical Research. As a part of the University of Cyprus, this core facility will collect over 16,500 donors and together with other partners (Medical University of Graz, Austria, Biobanking and BioMolecular Resources Research Infrastructure-European Research Infrastructure Consortium/BBMRI-ERIC, Austria; RTD TALOS Limited Cyprus) it will be responsible for completion of the Cyprus human genome project [66].

### 3.20. Malta

The Maltese Genome Project was founded as a part of the Malta BioBank, which is a member of BBMRI-ERIC. The main aim of the genome project is to obtain genome data from 1% of the Maltese population in relation to origins, mobility, epidemiology, pharmacogenomics and immunogenetics for gene discovery research [67].

### 3.21. Russia

The extreme diversity of the Russian population is one of the main causes of underrepresentation of genetic information in large worldwide datasets such as HapMap or 1KGP. The Genome Russia project (http://genomerussia.spbu.ru; accessed on 1 February 2022), launched by St. Petersburg State University and Dobzhansky Center for Genome Bioinformatics, is focusing on collection of samples from at least 3000 individuals from different parts of the Russian federation, whose ancestors are indigenous to the region for several generations. The sequencing data from this trio-design study will allow creating the database of medically relevant genomic variants characteristic to the Russian population, which would be the basis for developing the principles of the future personalized medicine [68]. A pilot study of the project used the WGS approach for analysis of genetic variability in a cohort of 264 samples obtained from 52 isolated populations across the Russian federation. The variant calling showed 8 million SNPs and 2 million indels per population and 4% of these SNPs were classified as novel when compared to dbSNP [69].

## 4. Discussion

Recent development of massive parallel sequencing technology launched plenty of both international and local GWAS studies, leading to better characterization of genetic variability among human populations. Current version of human genome assembly GRCh38/hg38 and its predecessors covered genetic variability of the local European population only briefly, and thus there is a strong need for further genomic data from local or native populations such as Finland, Iceland, Baltic and south European countries. From this point of view, it is not surprising that basically every further published national genome project from European country showed additional data not included in this assembly. In addition, those studies showed novel information about other aspects such as biobanking, GDPR, library preparation, sequencing workflow, utilization of novel data processing and data mining algorithms. Information about projects with published scientific results is summarized in Table 1. Further development of local genome projects in Europe should also bring ´1+ Million Genomes (+1MG)’ European initiative. The goal of 22 signatory EU countries is to obtain sequenced genomes from more than 1 million individuals by 2022 in order to create a framework that will cover analysis of genomic and health data both inside and across national boundaries in Europe. The methodical basis of the initiative is based on the Horizon 2020 project Beyond 1 Million Genomes (B1MG; https://b1mg-project.eu/), which is focused on infrastructure setup, legal and technical guidance, data standards and best practices to enable data access [70]. Another project associated with the +1MG initiative is the multi-country project called Genome of Europe. Together with B1MG it is focused on building a robust and high-quality European network of national genomic reference cohorts, representative of the European population. Connected via +1MG initiative, those individual datasets from the EU population will create a world-class European reference database for research and innovation of healthcare [71].

Taken together, the need for the completion of the genetic diversity map of human populations makes obvious that further local sequencing projects are still needed. Improvement of datasets and references introduced by previous large-scale sequencing initiatives such as 1KGP or HapMap is now more effective thanks to broader availability of WGS techniques. Genomic data provided by various countries world-wide from local sequencing projects should therefore lead to rapid improvement in the area of precision and/or personalized medicine and thus bring another important tool to the clinical diagnostics of the diseases.

## Figures and Tables

**Table 1:** Overview of basic characterizes of UK or European national genome projects with published scientific results.

Project	Country	Cohort Size	Year of Publishing	Library Preparation	Sequencing Technology	Website	Reference
UK10k	United Kingdom	3781	2013	Illumina pair-end (BGI, Sanger)	Illumina (BGI, Sanger)	https://www.uk10k.org/	[27]
deCODE Genetics	Iceland	2636	2015	TruSeq SBS	HiSeq, GAIIx	https://www.decode.com/research/	[36]
SweGen	Sweden	1000	2017	TruSeq PCR-free 2.0	HiSeq X	https://swefreq.nbis.se/dataset/SweGen	[38]
Sequencing Initiative Suomi in Finland (SISU)	Finnland	3000	2014	Agilent, Illumina, Roche	NA	http://www.sisuproject.fi/	[40]
Genome Denmark	Denmark	150	2017	Illumina	Illumina HiSeq2000	https://genome.au.dk/	[42]
Genome of Netherlands (GoNL)	Netherlands	769	2014	TruSeq 2.0, Nextera	HiSeq 2000	http://www.nlgenome.nl/	[53]
Italian Genome Reference Panel (IGRP1.0)	Italy	947	2020	Illumina	Illumina	https://www.iigm.it/site/	[57]
The Thousand Polish Genomes Project	Poland	1079	2021	TruSeq DNA PCR-free kit	NovaSeq 6000	https://www.genompolski.pl/	[61]
Genome Russia	Russia	3000	2018	TruSeq PCR free	HiSeq X, NovaSeq	http://genomerussia.spbu.ru/	[69]

## Data Availability

Not applicable.
